# Evaluation of the ileal digestibility and excreta retention of phosphorus for feed phosphates in broiler chickens and in Pekin ducks

**DOI:** 10.1016/j.psj.2022.101837

**Published:** 2022-03-10

**Authors:** H.Y. Wang, T.J. Applegate, K.Y. Zhang, G. Tian, X.M. Ding, S.P. Bai, J.P. Wang, L. Lv, Y. Xuan, H.W. Peng, S.X. Xu, Q.F. Zeng

**Affiliations:** ⁎ Institute of Animal Nutrition, Key Laboratory for Animal Disease-Resistance Nutrition of China, Ministry of Education, Sichuan Agricultural University, Chengdu, 611130, China; † Department of Poultry Science, University of Georgia, GA, 30602, USA; ‡ Guizhou Chanhen Chemical Corporation, Guizhou, 550500, China

**Keywords:** broilers, feed phosphate, meat duck, ileal phosphorus digestibility, phosphorus retention, the substitution method

## Abstract

The study aimed to determine the ileal phosphorus (**P**) digestibility (**iPD**) and the excreta P retention (**ePR**) of 5 monodicalcium phosphate (**MCP**) samples and 3 dicalcium phosphate (**DCP**) samples in broiler chickens and in Pekin ducks using the substitution method. A total of 720, 21-d-old Arbor Acres broiler chickens in experiment 1 and 720, 15-d-old Pekin ducks in experiment 2 were randomly allocated to 9 dietary treatments with 8 replicate cages (10 birds/cage) based on the similar mean body weight, respectively. The collection of excreta (for 72 h after a 3-d acclimation) and ileal digesta (after 6 d of feeding experimental diets) was done. The results showed the average iPD/ePR of MCP and DCP for broilers were 83.11%/74.52% and 75.34%/69.46% and for ducks were 79.37%/80.02% and 75.74%/76.44%, respectively. The iPD/ePR of MCP in broilers and the ePR of MCP in ducks were markedly higher (*P* < 0.05) than those of DCP. Our data suggest that using the substitution method to evaluate the bioavailability of feed phosphates has its own advantages; MCP has higher biological availability than DCP for broilers and ducks.

## INTRODUCTION

Phosphorus (**P**) has many biological functions in animal growth and maintaining health (Berndt and Kumar, 2009). Reduction of dietary nonphytate phosphorus (**nPP**) of broilers or meat ducks led to poor bone mineralization and thus impaired animal welfare or increased processing losses (Applegate and Angel, 2008; Xu et al., 2019). Thus, in commercial poultry production systems, using a large safety margin in diet formulation for supplemental P has resulted in a large amount of P excretion, which is becoming a severe environmental pollution issue (Liu et al., 2008). Meanwhile, nutritionist do not formulate diets with actual phosphorus availability or retention data but most assume that P is 100% available in inorganic phosphate (Leske and Coon, 2002). In fact, Coon et al. (2007) found that the excreta retention value of a reagent grade dicalcium phosphate (**DCP**) and 2 different defluorinated phosphate was only 82.99%, 76.34%, and 70.30%, respectively. Therefore, knowledge of absolute P digestibility and retention for feed phosphates in poultry is of economic and ecologic importance.

However, it is among debate within the scientific community as to which method is most effective and accurate for determining P digestibility and bioavailability of feed phosphates for poultry. World's Poultry Science Association (2013) recommended that the regression method is regarded as a standard method for determining the ileal digestibility and excreta retention of P in poultry. However, the regression method requires a basal diet and diets supplemented with at least two concentrations of a test P source, making it more expensive and laborious and the values of the ileal P digestibility (**iPD**) or the excreta P retention (**ePR**) can be affected by dietary nPP levels. An et al. (2020) used the direct method to determine the iPD and ePR of commercial feed phosphates in broilers and recommended that the direct method has some advantages in comparison with the regression method. The semi-purified diets, however, are used in the direct method are less practical and cause an abnormal growth or physiology for poultry. To our best of knowledge, no information was about the evaluation of the iPD and ePR of commercial feed phosphates using the substitution method in poultry.

Moreover, many researches have evaluated the bioavailability of P in different inorganic phosphates in broilers (Sullivan, 1996; Leske and coon, 1999; WPSA, 2013; An et al., 2020); but few have published information regarding the P digestibility and retention of different inorganic phosphates for meat ducks. Poultry and waterfowl have substantial physiological and physical differences in their digestive tract (Gabriel et al., 2008; Lu et al., 2011; Han et al., 2017) which results in the differences in nutrient digestibility and retention. Monocalcium phosphate (**MCP**) and DCP are the main forms of inorganic P sources used in poultry feed industry (Kleyn, 2013). Cordel et al. (2009) reported that feed phosphate is derived from phosphate rock, which is a nonrenewable resource, and current global reserves may be depleted in 50 to100 years. Extending and improving the database for P availability in feed ingredients, including variation within one ingredient, is therefore urgently needed and is one approach to address the problem (Rodehutscord et al., 2012). Therefore, the objectives of the present study were (1) to determine iPD and ePR of MCP (5 samples) and DCP (3 samples) in broilers and in Pekin ducks with the substitution method based on an ad libitum fed, practical diet assay, and (2) to make a limited comparison on the difference of iPD and ePR of different feed phosphates between broilers and ducks.

## MATERIALS AND METHODS

All the procedures used in the study were approved by the Institutional Animal Care and Use Committee of Sichuan Agricultural University (SAU-PND-2020-05).

### Experimental Design and Procedure

A total of 8 inorganic P sources tested were 5 commercial MCP samples and 3 commercial DCP samples. The analyzed total calcium (**Ca**), total P, fluorine (**F**), arsenic (**As**), lead (**Pb**), cadmium (**Cd**), chromium (**Cr**), and free phosphoric acid content as well as pH value of the 8 inorganic P sources are shown in Table 1. There was a total of 9 dietary treatments, including a basal diet and 8 test diets. The basal diet was formulated to meet or exceed the nutrient requirements of growing broilers or Pekin ducks recommended by NRC (1994), except for dietary nPP levels. The composition of the 100% basal diet for broilers or meat ducks with 0.5% Titanium (**TiO_2_**) as an indigestible marker are presented in Table 2, respectively. The tested MCP and DCP diets included 85% basal diet and 15% inorganic phosphate mixed corn starch, which all contained 0.42% nPP levels based on the theory of the substitution method (Zhang et al., 2020). Briefly, to keep all test diets contained 0.42% nPP levels, we first calculate the nPP levels in the 85% basal diets (e.g., dietary 0.30% nPP × 85% = 0.255%) and the other 15% space should provide 0.165% nPP (e.g., 0.42%–0.255%=0.165%) or should contain 1.1% nPP (e.g., 0.165%/15% = 1.1%). After that, we determine the ratio of feed phosphate to corn starch in the 15% space based on the analyzed P content of each feed phosphates. The substitution ratio was the total P content in 15% space/the total P content in the corresponding 100% test diet. The analyzed total Ca and total P as well as Ca to P ratio in 11 diets are shown in Table 3.

**Table 1:** Chemical characteristics of feed phosphates based on analyzed value.

Sample	Total P, %	Soluble P, %	Total Ca, %	F, %	pH Value	As,mg/kg	Pb, mg/kg	Cd,mg/kg	Cr, mg/kg	Free H_3_PO_4,_ %
MCP 1	22.14	20.09	13.52	0.13	3.44	2.57	3.77	0.37	22.30	10.78
MCP 2	22.41	20.31	13.67	0.13	3.55	12.47	4.37	0.68	29.30	7.78
MCP 3	22.43	20.39	13.36	0.13	3.65	4.27	3.93	1.48	26.30	6.35
MCP 4	22.23	20.13	12.59	0.21	3.76	12.77	6.17	0.69	44.03	3.94
MCP 5	22.98	20.30	14.20	0.12	3.66	8.67	3.90	0.40	28.23	5.53
DCP 1	17.31	2.05	21.61	0.15	7.85	16.33	3.17	0.54	7.80	-
DCP 2	17.21	2.19	21.01	0.15	7.66	10.60	3.50	0.91	15.23	-
DCP 3	18.33	1.98	23.45	0.18	7.83	9.23	5.53	1.80	4.67	-

P: phosphorus; Ca: calcium; F: fluorine; As: arsenic; Pb: lead; Cd: cadmium; Cr: chromium; -: means no detect; MCP: monocalcium phosphate; DCP: dicalcium phosphate.

**Table 2:** The composition and nutrient levels of the basal diets.

Items	Broilers (21 to 27 d of age)	Ducks (15 to 21d of age)
Ingredients, %		
Corn starch	9.85	9.85
Corn	43.50	55.00
Soybean oil	0.57	-
Corn gluten meal	9.25	6.00
Soybean meal	32.00	24.30
*L*-Lysine·HCl	0.366	0.366
*L*-Threonine	0.065	0.065
Tryptophan	0.00	0.042
*DL*-Methionine	0.08	0.08
Calcium carbonate	1.55	1.42
Dicalcium phosphate	1.03	1.08
Sodium chloride	0.35	0.35
Choline chloride	0.15	0.15
Vitamin premix^1^	0.03	0.03
Mineral premix^2^	0.50	0.50
Titanium dioxide	0.50	0.50
Zeolite	0.209	0.267
Total	100.00	100.00
Calculated nutrient levels, %		
ME(MJ/kg)	12.14	12.13
Crude protein	23.00	19.05
Calcium	0.90	0.85
Total phosphorus	0.53	0.51
Nonphytate phosphorous	0.30	0.30
Calcium to Phosphorus ratios	1.70	1.67
Lysine	1.47	1.06
Methionine	0.48	0.40
Threonine	0.80	0.69
Tryptophan	0.22	0.22

1 Vitamin premix provided the vitamin composition and content for per kg of diets: Vitamin A 12, 000 IU; Vitamin D_3_ 2, 000 IU; Vitamin E 7.0 mg; Vitamin K_3_ 4.5 mg; Vitamin B_1_ 3 mg; Vitamin B_2_ 7 mg; Vitamin B_6_3 mg; VitaminB_12_ 0.01 mg; Calcium pantothenate 15 mg; folic acid 1.8 mg; Biotin 0.22 mg; Nicotinic acid 79 mg; Vitamin C 100 mg.

2 Mineral premix provides following per kg of the diet: Fe(FeSO_4_·H_2_O) 80 mg; Cu(CuSO_4_·5H_2_O) 10 mg; Mn (MnSO_4_·H_2_O) 100 mg; Zn(ZnSO_4_·H_2_O) 60 mg; I (KI) 0.45 mg; Se (Na_2_SeO_3_) 0.3 mg.

**Table 3:** Analyzed dietary calcium and total phosphorus content.

Tested diets	Broiler chickens			Meat ducks		
	Total Ca (g/kg)	Total P (g/kg)	Ca:P ratios	Total Ca (g/kg)	Total P (g/kg)	Ca:P ratios
Basal	10.2	4.5	2.27	10.5	4.1	2.56
MCP1	9.9	5.4	1.83	8.5	5.2	1.63
MCP2	10.7	5.3	2.02	10.0	5.3	1.88
MCP3	10.3	5.8	1.78	9.7	5.4	1.80
MCP4	11.6	6.1	1.90	10.2	6.5	1.57
MCP5	10.1	5.6	1.80	8.9	5.1	1.75
DCP1	10.7	5.4	1.98	10.8	5.4	2.00
DCP2	11.3	5.7	1.98	11.2	5.4	2.07
DCP3	10.9	5.5	1.98	10.1	5.1	1.98

P: phosphorus; Ca: calcium; MCP: monocalcium phosphate; DCP: dicalcium phosphate.

### Experiment 1

In order to evaluate the iPD and ePR for inorganic phosphate sources in broilers, a total of 800 one-day-old Arbor Acres male broiler chickens were obtained from a local hatchery and housed in electrically heated, thermostatically controlled stainless cages coated with plastic (100 by 100 by 50 cm). Feed and tap water were available ad libitum. All birds during 1 to 20 d of age were fed the same diet containing (per kg) 210 g of CP, 12.33 MJ of ME, 8.3 g of Ca, and 4.5 g nPP. At 21 d of age, 720 birds were weighed and randomly allocated to 9 dietary treatments with 8 replicate cages (10 birds/cage) based on the similar mean body weight. From 21 to 27 d of age, experimental diets and water were available for ad libitum consumption. All experimental diets were pelleted with a diameter 2.5 mm. After acclimation for 3 d, on d 24 at 0800 h, excreta were collected for continued 3 d (72 h; collected per 2 h and pooled by cage) to determine the excreta P retention based on the study of Liu et al. (2013). On d 27 at 0800 h, birds were euthanized using carbon dioxide (**CO_2_**) and the digesta from the terminal two-thirds of ileum were collected by gently squeezing the contents of the ileum into sample bags according to the procedure of Rodehutscord et al. (2012). Digesta from broilers within a cage were pooled and frozen immediately after collection and subsequently freeze-dried. The dried ileal digesta and excreta were stored in airtight bags at –4°C until needed for chemical analysis.

### Experiment 2

In order to evaluate the iPD and ePR for inorganic phosphate sources in Pekin ducks, a total of 800 one-day old Pekin male ducklings were obtained from a local hatchery and housed in an environmentally controlled room. Feed and tap water were also available ad libitum. All ducks during 1 to 14 d of age were fed the same diet containing (per kg) 195 g of CP, 11.91 MJ of ME, 8.0 g of Ca, and 4.0 g nPP. At 15 d of age, 720 birds were weighed and randomly allocated to 9 dietary treatments with 8 replicate cages (10 birds/cage) based on the similar mean body weight. From 15 to 21 d of age, the experimental diets and water were available for ad libitum consumption. After acclimation for 3 d, on d 18 at 0800 h, excreta were collected for continued 3 d (72 h; collected per 2 h and pooled by cage) to determine the excreta P retention. On d 21 at 0800 h, birds were euthanized using CO_2_ and the digesta from the terminal two-thirds of ileum were collected by gently squeezing the contents of the ileum into sample bags. The other management and test procedure were similar to those described for Experiment 1.

### Chemical Analysis

The concentrations of F, AS, Pb, Cd, and Cr were determined using inductively coupled plasma-mass spectrometry as described by Li et al. (2011). Dried excreta and digesta samples were ground through a 0.45-mm sieve using a grinding mill to facilitate analyses (Adeola et al., 1997). Diets, digesta, and fecal samples were analyzed for DM contents (ISO 6496, 1998a). Concentrations of total P concentrations and soluble P in inorganic P sources, diets, and fecal samples were determined using a spectrophotometer (ISO 11885, 1998b). Titanium content of experimental diets, ileal digesta, and excreta were determined by UV spectroscopy (Zhang et al., 2020).

### Calculations and Statistical Analyses

The ileal digestibility (%) and excreta retention (%) of inorganic P sources were individually calculated according to the following equations:

$$\text{Ileal}\;\text{digestibility}\;\left(\%\right)\text{of}\;P\;\text{in}\;\text{diets}\;=\;\left(1-\frac{Pi\times Td}{Pd\times Ti}\right)\times \;100;$$

in which Pi is the total P (mg) in ileum, Td is the TiO_2_ in diets, Pd is the total P in diets, Ti is the TiO_2_ in ileum.

$$\text{Excreta}\;\text{retention}\;\left(\%\right)\text{of}\;P\;\text{in}\;\text{diets}\;=\;\left(1-\frac{Pe\times Td}{Pd\times Te}\right)\times \;100;$$

in which Pe is the total P (mg) in excreta, Te is the TiO_2_ in excreta.

Ileal digestibility (%) or Excreta availability (%) of P in inorganic phosphate

=B−(B−A)/F

where B is the ileal digestibility (%) or excreta retention (%) of P in basal diet; A is the ileal digestibility (%) or excreta retention (%) of P in assay diet; F is the proportion of P from 15% space to it from the assay diet, that is, F was the total P content in 15% space/ the total P content in the corresponding 100% test diet.

The ileal digestibility (%) or excreta retention (%) of P in the same inorganic phosphate were analyzed by one-way ANOVA using the GLM procedure of SAS (SAS Institute Inc., Cary, NC). Differences among means were tested by the least significant difference (**LSD**) test. The cage served as the experimental unit for all statistical analyses, and the *P ≤* 0.05 was considered to be statistically significant. Differences of P digestibility or retention between broiler chickens and Pekin ducks were evaluated using a 2-tailed unpaired *t*-test or the Mann-Whitney *U* test for normally or non-normally distributed datasets, respectively.

## RESULTS

### The Chemical Characters in 8 Feed Phosphates Samples

As shown in Table 1, the total P content in 5 MCP samples and 3 DCP samples were from 22.14 to 22.98% and from 17.21 to 18.33%, respectively. The pH values in MCP were the lower than that in DCP. Correspondingly, the content of free phosphoric acid was the higher in MCP, and no detection in DCP. But the content of free phosphoric acid in MCP 1 was 10.78% which was higher than that in other 4 MCP samples. The content of F and heavy metals (e.g., AS, Pb, Cd, and Cr) in all samples was below the limited standard according to feed regulations in China.

### The Analyzed Ca and P Content in Basal Diets and All Tested Diets

Table 3 shows the Ca and P content as well as Ca to P ratios in 9 diets. The basal diet of broilers and ducks had a higher Ca to P ratio, which were similar with the calculated value in the basal diets. The Ca to P ratios in 8 diets ranged from 1.78 to 2.02 in broiler's tested diets and ranged from 1.57 to 2.07 in duck's tested diets.

### The Ileal P Digestibility and Excreta P Retention of MCP and DCP in Broilers

As shown in Table 4, for broilers, the iPD of 5 MCP samples was 83.11% (from 76.18% to 86.41%) and the ePR was 74.52% (from 72.03% to 78.78%); the value of iPD was higher than that of ePR. The iPD and ePR in broilers had no marked difference (*P >* 0.05) among 5 MCP samples.

**Table 4:** The ileal digestibility and excreta retention of phosphorus from feed monocalcium phosphates and dicalcium phosphates in broiler chickens (Exp1),^1^ %.

Items	Broiler chickens	
	Digestibility of P	Retention of P
5 MCP samples		
MCP1	85.48	72.03
MCP2	82.62	75.36
MCP3	86.41	72.67
MCP4	84.86	78.78
MCP5	76.18	73.76
SEM	3.15	3.8
*P-value*	0.17	0.74
Mean	83.11	74.52
CV	4.95	3.62
3 DCP samples		
DCP1	67.83^b^	62.84^b^
DCP2	78.28^a^	71.32^a^
DCP3	79.91^a^	74.22^a^
SEM	2.55	3.13
*P-value*	0.01	0.04
Mean	75.34	69.46
CV	8.70	8.51

1 Means represent 8 cages of birds, 10 birds per cage.

a,b Means in columns with no comment superscripts are significantly different under each phosphate(*P* < 0.05). P: phosphorus; MCP: monocalcium phosphate; DCP: dicalcium phosphate; CV: coefficient of variation.

The iPD and ePR of DCP1 was significantly lower than those of DCP2 or 3 in broilers (*P* < 0.05). Moreover, for broilers, the iPD of DCP was 75.34% (from 67.83% to 79.91%) and the ePR was 69.46% (from 62.84 % to 74.22%); the value of iPD was also higher than that of the ePR.

### The Ileal P Digestibility and Excreta P Retention of MCP and DCP in Ducks

As shown in Table 5, for ducks, the iPD of 5 MCP samples was 79.37% (from 73.31% to 90.05%) and the ePR was 80.02% (from 75.61% to 82.77%); the value of iPD was close to that of the ePR. Only the iPD in 5 MCP samples presented a significant difference (*P* < 0.05) in meat ducks. The iPD of MCP4 was higher (*P* < 0.05) than that of MCP1, MCP2 and MCP3 in ducks. However, the ePR in ducks had no marked difference (*P >* 0.05) among 5 MCP samples.

**Table 5:** The ileal digestibility and excreta retention of phosphorus from feed monocalcium phosphates and dicalcium phosphates in meat ducks (Exp2)^1^, %.

Items	Meat ducks	
	Digestibility of P	Retention of P
5 MCP samples		
MCP1	74.32^b^	80.78
MCP2	78.82^b^	78.54
MCP3	73.31^b^	75.61
MCP4	90.05^a^	82.39
MCP5	80.34^ab^	82.77
SEM	3.82	3.95
P-Value	0.03	0.69
Mean	79.37	80.02
CV	8.39	3.72
3DCP samples		
DCP1	75.39	77.07
DCP2	74.91	78.60
DCP3	76.93	73.64
SEM	3.69	3.46
P-value	0.92	0.59
Mean	75.74	76.44
CV	1.39	3.32

1 Means represent 8 cages of birds, 10 birds per cage.

a,b Means in columns with no comment superscripts are significantly different under each phosphate(*P* < 0.05). P: phosphorus; MCP: monocalcium phosphate;DCP: dicalcium phosphate; CV: coefficient of variation.

The iPD of 3 DCP samples for ducks was 75.74% (from 74.91% to 76.93%) and the ePR was 76.44% (from 73.64% to 78.60%); the value of the iPD was also close to that of the ePR.

## DISCUSSION

It is difficult to precisely evaluate the bioavailability of feed phosphates in poultry due to different experimental methodology (e.g., substitution, direct, and regression), the nPP concentrations and Ca: P ratios of experimental diets, age and lines of poultry, feeding period, and particle size Shastak and Rodehutscord (2013) suggested the ileal digestibility and excreta retention are the most appropriate criteria for evaluating P sources in poultry and still need the development of different approaches to determine P availability. Thus, in the present study, we first tried to use the substitution method to evaluate the ileal P digestibility (**iPD**) and excreta P retention (ePR) of different feed phosphates in broilers and ducks, respectively. The values of iPD and ePR of different feed phosphates were close to those in the studies of Shastak et al. (2012); Leske and Coon (2002); Trairatapiwan et al. (2018); An et al. (2020) and Ketels and De Groote (1988). Shastak et al. (2012) and Leske and Coon (2002) found that the P retention of anhydrous monosodium phosphate (from 70% to 81%) and of reagent-grade MCP (from 59% to 98% depending on dietary nPP levels. Trairatapiwan et al. (2018) showed that the ileal P digestibility of MCP and DCP (from bone) were 64.6% and 69.3%, respectively. An et al. (2020) determined that the iPD and ePR of commercial MCP and DCP were 86.7%/64.0% and 76.2%/57.4% in broilers aged from 15 to 18d used the direct method. Ketels and De Groote (1988) found that the ileal digestibility of P from anhydrous DCP and DCP × H_2_O in 3-wk-old broilers to be 67% and 73%, respectively. These above results suggested that the substitution method is also a good method to evaluate the P bioavailability of feed phosphates in poultry.

In the present study, moreover, the results showed the P digestibility and retention of MCP were higher than those of DCP in broilers or in ducks. These values agree with the study of Bikker et al. (2016), which showed the ileal P digestibility of MCP (78.3%) > DCP (59.0%) in male broiler chickens. An et al. (2020) also showed that the P digestibility and retention of MCP (86.7%/64.0%) > DCP (76.2%/57.4%) in broilers. De Groote and Huyghebaert (1997) found that the apparent P retention with the pelleted diet was on average 78.1%, 74.2%, and 63.6% respectively for MCP, DCP, and anhydrous DCP, and the effect of Ca (9.1 vs. 10.5g/kg) was not significant. Axe (1998) suggested that the differences from the biological availability or utilization of different phosphates can be attributed to type, source, and particle size of phosphates. The main differences of chemical characteristics in MCP and DCP were the solubility, pH values, and free phosphoric acid content in the present study. We conjectured that the pH values and free phosphoric acid content in feed phosphates maybe 2 key factors to affect their P digestibility and retention. Vieira et al. (2017) found that supplemented of acidifier could improve performance and bone mineralization of broilers by increasing the P digestibility and retention. As noted above, MCP is more biologically available feed phosphate source than DCP for poultry. This agrees with a study of Lamp et al. (2020), which found that broilers fed with MCP demonstrated increased live weight gain, tibia ash (mg/chick), and mineral digestibility compared with birds fed with DCP when diets were formulated to similar NPP content and Ca: P.

Interestingly, we found that the iPD were higher than the ePR of MCP and DCP in broilers. The differences of iPD and ePR lied in P excretion with urine or postileal absorption and secretion of P by postileal fermentation (Ravindran et al., 1999). Manangi and Coon (2006) used 40- and 50-d-old colostomized broilers to study the effect of different dietary nPP levels on urinary P excretion, which found that urinary excretion of P remained constant and very low from 0.08% to 0.28% dietary nPP in 40-d-old birds (6.0 ± 3.2 mg/d) and 0.08% to 0.21% dietary nPP in 50-d-old birds (1.9 ± 3.5 mg/d). If the urine had been relevant for P excretion, then the value of ePR should have been lower than that of iPD, which was the case in the current study. Biehl and Baker (1997) also found no differences in tibia ash between cecectomized and intact chicks. This suggests that inorganic phosphate, although released from inositol phosphates by microbial activity in the ceca, was not absorbed (Kerr et al., 2000). The reason lied in the loss of urine P.

## CONCLUSIONS

In conclusion, the substitution method proved to give reasonable values of iPD and ePR of feed phosphates in poultry. The iPD and ePR of MCP were more biological available than these of DCP, which suggests MCP is a better feed phosphate for poultry. For broiler chicks, the iPD was higher that the ePR of MCP and DCP, and it is more reasonable to use the ileal P digestibility of feed phosphates when formulating diets in broiler chicks.
